# Univariate comparison of performance of different cerebrovascular reactivity indices for outcome association in adult TBI: a CENTER-TBI study

**DOI:** 10.1007/s00701-019-03844-1

**Published:** 2019-03-15

**Authors:** Frederick A. Zeiler, Ari Ercole, Manuel Cabeleira, Tommaso Zoerle, Nino Stocchetti, David K. Menon, Peter Smielewski, Marek Czosnyka, CENTER-TBI High Resolution Sub-Study Participants and Investigators, Audny Anke, Ronny Beer, Bo-Michael Bellander, Andras Buki, Giorgio Chevallard, Arturo Chieregato, Giuseppe Citerio, Endre Czeiter, Bart Depreitere, George Eapen, Shirin Frisvold, Raimund Helbok, Stefan Jankowski, Daniel Kondziella, Lars-Owe Koskinen, Geert Meyfroidt, Kirsten Moeller, David Nelson, Anna Piippo-Karjalainen, Andreea Radoi, Arminas Ragauskas, Rahul Raj, Jonathan Rhodes, Saulius Rocka, Rolf Rossaint, Juan Sahuquillo, Oliver Sakowitz, Ana Stevanovic, Nina Sundström, Riikka Takala, Tomas Tamosuitis, Olli Tenovuo, Peter Vajkoczy, Alessia Vargiolu, Rimantas Vilcinis, Stefa Wolf, Alexander Younsi

**Affiliations:** 1 Division of Anaesthesia, Addenbrooke’s Hospital, University of Cambridge, Cambridge, UK; 2 Department of Surgery, Rady Faculty of Health Sciences, University of Manitoba, Winnipeg, MB R3A 1R9 Canada; 3 Clinician Investigator Program, Rady Faculty of Health Science, University of Manitoba, Winnipeg, MB Canada; 4 Brain Physics Laboratory, Division of Neurosurgery, Dept of Clinical Neurosciences, Addenbrooke’s Hospital, University of Cambridge, Cambridge, UK; 5 Neuro ICU Fondazione IRCCS Cà Granda Ospedale Maggiore Policlinico, Milan, Italy; 6 Department of physiopathology and transplantation, Milan University, Milan, Italy; 7 Neurosciences Critical Care Unit, Addenbrooke’s Hospital, Cambridge, England; 8 Queens’ College, Cambridge, England; 9 National Institute for Health Research, London, UK; 10 Institute of Electronic Systems, Warsaw University of Technology, Warsaw, Poland

**Keywords:** Autoregulation, Cerebrovascular reactivity, ICP indices, Outcome analysis

## Abstract

**Background:**

Monitoring cerebrovascular reactivity in adult traumatic brain injury (TBI) has been linked to global patient outcome. Three intra-cranial pressure (ICP)-derived indices have been described. It is unknown which index is superior for outcome association in TBI outside previous single-center evaluations. The goal of this study is to evaluate indices for 6- to 12-month outcome association using uniform data harvested in multiple centers.

**Methods:**

Using the prospectively collected data from the Collaborative European NeuroTrauma Effectiveness Research in TBI (CENTER-TBI) study, the following indices of cerebrovascular reactivity were derived: PRx (correlation between ICP and mean arterial pressure (MAP)), PAx (correlation between pulse amplitude of ICP (AMP) and MAP), and RAC (correlation between AMP and cerebral perfusion pressure (CPP)). Univariate logistic regression models were created to assess the association between vascular reactivity indices with global dichotomized outcome at 6 to 12 months, as assessed by Glasgow Outcome Score–Extended (GOSE). Models were compared via area under the receiver operating curve (AUC) and Delong’s test.

**Results:**

Two separate patient groups from this cohort were assessed: the total population with available data (*n* = 204) and only those without decompressive craniectomy (*n* = 159), with identical results. PRx, PAx, and RAC perform similar in outcome association for both dichotomized outcomes, alive/dead and favorable/unfavorable, with RAC trending towards higher AUC values. There were statistically higher mean values for the index, % time above threshold, and hourly dose above threshold for each of PRx, PAx, and RAC in those patients with poor outcomes.

**Conclusions:**

PRx, PAx, and RAC appear similar in their associations with 6- to 12-month outcome in moderate/severe adult TBI, with RAC showing tendency to achieve stronger associations. Further work is required to determine the role for each of these cerebrovascular indices in monitoring of TBI patients.

**Electronic supplementary material:**

The online version of this article (10.1007/s00701-019-03844-1) contains supplementary material, which is available to authorized users.

## Introduction

Various continuous indices for monitoring cerebrovascular reactivity exist[2, 7, 20]. These indices are derived from the different cranial multi-modal monitoring (MMM) devices employed for the monitoring of critically ill patients. In the traumatic brain injury (TBI) literature, the indices derived from invasive intra-cranial pressure (ICP) monitoring are the most widely described, with pressure reactivity index (PRx—the correlation between ICP and mean arterial pressure (MAP)) considered a standard, and widely applied, index for monitory cerebrovascular reactivity in adult TBI [1, 3, 6–9, 13, 16, 18, 20, 22].

The literature supports a strong association between PRx and global patient outcome in adult TBI, [7, 18] with experimental literature validating it as a measure of cerebral autoregulation [4, 19, 23]. However, two other ICP-derived indices exist: pulse amplitude index—PAx (correlation between pulse amplitude of ICP (AMP) and MAP) [2] and RAC (correlation (R) between AMP (A) and cerebral perfusion pressure (CPP)) [21]. Both of these newer indices have also been validated as measures of cerebral autoregulation in experimental animal models [19, 23]. However, it must be noted that all three of these ICP-derived indices have only been validated against the lower limit of autoregulation, [4, 19, 23] with literature validating their ability to detect the upper limit currently unavailable.

Some preliminary literature based on single-center retrospective study supports the superiority of RAC in its association with 6-month global outcome, over both PRx and PAx, in adult TBI [22]. The goal of this multi-center study, using the high-resolution intensive care unit (ICU) cohort from the Collaborative European Neuro Trauma Effectiveness Research in TBI (CENTER-TBI) study, [15] was to determine which ICP index of cerebrovascular reactivity is superior in its association with 6- to 12-month global outcome in adult TBI, evaluating raw index values and derived parameters.

## Methods

### Patient population

All patients from the multi-center CENTER-TBI high-resolution ICU cohort were included for this study. These patients were prospectively recruited during the periods of January 2015 to December 2017. A total of 21 centers in the European Union (EU) recruited patients for this cohort. All patients were admitted to ICU for their TBI during the course of the study, with high-frequency digital signals recorded from their ICU monitors during the course of their ICU stay. All patients predominantly suffered from moderate to severe TBI (moderate = Glasgow Coma Score (GCS) 9 to 12 and severe = GCS of 8 or less). A minority of patients suffered from non-severe TBI, with subsequent early deterioration leading to ICU admission for care and monitoring. All patients in this cohort had invasive ICP monitoring conducted in accordance with the BTF guidelines [5].

#### Ethics

Data used in these analyses were collected as part of the CENTER-TBI study which had individual national or local regulatory approval; the UK ethics approval is provided as an exemplar: (IRAS No: 150943; REC 14/SC/1370). The CENTER-TBI study (EC grant 602150) has been conducted in accordance with all relevant laws of the EU if directly applicable or if direct effect and all relevant laws of the country where the recruiting sites were located, including but not limited to, the relevant privacy and data protection laws and regulations (the “Privacy Law”), the relevant laws and regulations on the use of human materials, and all relevant guidance relating to clinical studies from time to time in force including, but not limited to, the ICH Harmonised Tripartite Guideline for Good Clinical Practice (CPMP/ICH/135/95) (“ICH GCP”) and the World Medical Association Declaration of Helsinki entitled “Ethical Principles for Medical Research Involving Human Subjects”. Informed consent by the patients and/or the legal representative/next of kin was obtained, accordingly to the local legislations, for all patients recruited in the Core Dataset of CENTER-TBI and documented in the e-CRF.

### Data collection

As part of recruitment to the multi-center high-resolution ICU cohort of CENTER-TBI, [15] all patients had demographics prospectively recorded. Similarly, all patients had high-frequency digital signals from ICU monitoring recorded throughout their ICU stay, with the goal of initiating recording within 24 h of injury. All digital ICU signals were further processed (see “Signal acquisition” and “Signal processing”). For the purpose of this study, the following admission demographic variables were collected: age, sex, and admission Glasgow Coma Scale (GCS—total and motor). Data was accessed on Sept 16th, 2018 via Opal database software [10].

### Signal acquisition

Arterial blood pressure (ABP) was obtained through either radial or femoral arterial lines connected to pressure transducers (Baxter Healthcare Corp. CardioVascular Group, Irvine, CA). ICP was acquired via an intra-parenchymal strain gauge probe (Codman ICP MicroSensor; Codman & Shurtleff Inc., Raynham, MA), parenchymal fiber optic pressure sensor (Camino ICP Monitor, Integra Life Sciences, Plainsboro, NJ, United States; https://www.integralife.com/), or external ventricular drain. All signals were recorded using digital data transfer or digitized via an A/D converter (DT9801; Data Translation, Marlboro, MA), where appropriate, sampled at frequency of 100 Hz (Hz) or higher, using the ICM+ software (Cambridge Enterprise Ltd., Cambridge, UK, http://icmplus.neurosurg.cam.ac.uk) or Moberg CNS Monitor (Moberg Research Inc., Ambler, PA, USA) or a combination of both. Signal artifacts were removed using both manual and automated methods prior to further processing or analysis.

Physiologic signals were recorded throughout the duration of ICP monitoring for the patients, with initiation of recording within 24 h of the injury. For the patients undergoing decompressive craniectomy, there were 45 in this cohort. Seventeen patients had secondary decompressive craniectomies (i.e., for refractory ICP), and thus had recordings both pre- and post-craniectomy. The remaining 28 underwent primary craniectomies near the time of admission, and thus only had physiologic signals recorded for the periods after craniectomy.

### Signal processing

Post-acquisition processing of the above signals was conducted using ICM+. CPP was determined as CPP = MAP–ICP. AMP was determined by calculating the fundamental Fourier amplitude of the ICP pulse waveforms over a 10 s window, updated every 10 s. Ten-second moving averages (updated every 10 s to avoid data overlap) were calculated for all recorded signals: ICP, ABP (which produced MAP), AMP, and CPP. This 10-s moving average filter is applied to the raw signals so as to decimate signal frequency into the range appropriate for vasogenic slow-wave evaluation (i.e., 0.05 to 0.005 Hz) [11, 12].

Continuous indices of cerebrovascular reactivity were derived via the moving correlation coefficient between 30 consecutive 10 s mean windows (i.e., vasogenic slow-wave fluctuations) of the parent signals, updated every minute. PRx was derived via the correlation between slow-wave fluctuations (i.e., 0.05 to 0.005 Hz) in ICP and MAP [7, 11, 12]. PAx was derived via the correlation between slow-wave fluctuations in AMP and MAP. RAC was derived via the correlation between slow-wave fluctuations in AMP and CPP. The focus on slow-wave frequency range (i.e., 0.05 to 0.005 Hz) is conducted to evaluate the vasogenic responses in the cerebrovascular system, with this frequency range validated in the literature to provide the optimal discriminatory frequency for phase-shift between signals [11, 12].

PRx has been thoroughly described in the TBI literature, with numerous publications to date [7, 16, 18, 20, 22]. PAx has been sparingly described and has displayed potential superiority over PRx in patients with persistently low ICP throughout their ICU stay [2, 22]. RAC is a newly described index which provides some suggestion of stronger association with 6-month outcome, compared to PRx and PAx [21, 22]. In general, these moving correlation coefficients between slow-wave fluctuations (i.e., frequency range of 0.05 to 0.005 Hz) [11, 12] in a measure of pulsatile cerebral blood volume, such as ICP or AMP, and a driving pressure to blood flow, such as MAP or CPP, provide information regarding cerebrovascular reactivity, with all three having some validation in experimental models of arterial hypotension [4, 23] and/or intra-cranial hypertension [19] as measures of the lower limit of autoregulation. These indices range from + 1.0, which typically denotes severely impaired cerebral autoregulation, to − 1.0, denoting intact autoregulation [20]. Various critical thresholds associated with 6-month outcome for each index have been described in the clinical literature [18, 22].

Data was provided in minute-by-minute comma separated variable sheets for the entire duration of ICP recording for each patient. The median recording duration for the included patients was 136.0 h (IQR: 90.2 to 182.3 h).

### Data processing

Grand mean values of all physiologic variables were calculated per patient. In addition, the following post-ICM+ processing of this physiologic data occurred in R (R Core Team (2016). R: A language and environment for statistical computing. R Foundation for Statistical Computing, Vienna, Austria. URL https://www.R-project.org/):

#### Cerebrovascular reactivity indices

- PRx—For each patient, the % of time spent above the following clinically defined thresholds was calculated across the entire recording period: 0, + 0.25, + 0.35. In addition, mean hourly dose of PRx above each of these thresholds was calculated.
- PAx—for each patient, the % of time spent above the following clinically defined thresholds was calculated across the entire recording period: 0, + 0.25. In addition, mean hourly dose of PAx above each of these thresholds was calculated.

c. RAC—For each patient, the % of time spent above the following clinically defined thresholds was calculated across the entire recording period: − 0.10, − 0.05. In addition, mean hourly dose of RAC above each of these thresholds was calculated.

### Statistics

All statistical analysis was conducted using R (R Core Team (2016). R: A language and environment for statistical computing. R Foundation for Statistical Computing, Vienna, Austria. URL https://www.R-project.org/) and XLSTAT (Addinsoft, New York, NY; https://www.xlstat.com/en/) add-on package to Microsoft Excel (Microsoft Office 15, Version 16.0.7369.1323). Normality of continuous variables was assessed via Shapiro-Wilks test. For all testing described within, the alpha was set at 0.05 for significance.

Despite GOSE being collected at both 6- and 12-month post-injury in this cohort of patients, there was missing data present in both categories of outcome. At the time point of data access for this study (Sept 2018), imputed global outcomes were not available for this cohort and are currently still to focus of a separate ongoing larger project on data imputation in CENTER-TBI, including the small number of high-resolution patients and the larger non-high-resolution ICU cohorts. Thus, we combined GOSE scores from both 6 and 12 months in order to provide a “6 to 12 Month” GOSE. For patients where GOSE was reported for both 6 and 12 months, the later GOSE score was selected for analysis. GOSE was then dichotomized into the following categories: (a) alive (GOSE 2 to 8) vs. dead (GOSE 1) and (b) favorable (GOSE 5 to 8) vs. unfavorable (GOSE 4 or less). Demographics and physiologic variables were compared between each dichotomized group, via *t* test, Mann-U, and Chi-square testing where appropriate.

Univariate logistic regression (ULR) was conducted, comparing each ICP cerebrovascular reactivity index variable (i.e., mean index value, % time above threshold, and mean hourly dose above threshold) to both dichotomized outcomes, assessing superiority via AUC and Delong’s test. Multi-variable models were not created in this study, as the focus was on univariate association with global outcome, in order to provide some validation for previous retrospective single-center work on ICP-derived indices of cerebrovascular reactivity [18, 22] and inform future planned multi-variable analysis.

## Results

### Patient population

At the time of this analysis, a total of 204 patients from the CENTER-TBI high-resolution ICU cohort had complete data sets, including: 6- to 12-month GOSE and high-frequency physiologic signals containing at least ICP and ABP for ICP cerebrovascular index derivation. A total of 159 patients did not undergo decompressive craniectomy (DC). The analysis was conducted in both the total population (*n* = 204) and the non-DC cohort (*n* = 159), yielding similar results. The patient demographics for the entire cohort can be found summarized in Table 1. Mann-U and Chi-square testing comparing various variables between the dichotomized outcome groups can be seen in Tables 2 and 3. Furthermore, the non-DC patient cohort demographic and comparison between groups can be found in Appendix A of the supplementary materials.

**Table 1:** Patient demographics–total population

		Median (IQR)
Number of patients		204
Age (years)		47.5 (29 to 62.3))
Sex	Male	163
	Female	41
Admission GCS (total)		7 (4 to 13)
Admission GCS motor		4 (2 to 6)
Duration of high-frequency cranial physiologic recording (hours)		136.0 (90.2 to 182.3)
ICP (mmHg)		12.2 (9.3 to 15.4)
CPP (mmHg)		70.9 (64.4 to 76.8)
% Time with ICP > 20 mmHg		4.7 (0.9 to 15.7)
% Time with ICP > 22 mmHg		2.8 (0.5 to 9.7)
Mean PRx		0.038 (− 0.071 to 0.164)
Mean PAx		− 0.030 (− 0.169 to 0.093)
Mean RAC		− 0.296 (− 0.484 to − 0.129)
% Time with PRx > 0		52.5 (40.2 to 67.8)
% Time with PRx > + 0.25		28.1 (19.1 to 41.4)
% Time with PRx > + 0.35		21.1 (14.0 to 31.2)
% Time with PAx > 0		45.8 (30.8 to 62.0)
% Time with PAx > + 0.25		21.0 (12.4 to 35.4)
% Time with RAC > − 0.10		27.0 (14.0 to 44.9)
% Time with RAC > − 0.05		22.5 (11.8 to 39.9)
Mean hourly dose of PRx > 0		8.9 (6.3 tot 13.0)
Mean hourly dose of PRx > + 0.25		3.7 (2.5 to 5.8)
Mean hourly dose of PRx > + 0.35		2.5 (1.5 to 3.9)
Mean hourly dose of PAx > 0		6.4 (4.0 to 10.2)
Mean hourly dose of PAx > + 0.25		2.3 (1.2 to 3.9)
Mean hourly dose of RAC > − 0.10		3.6 (1.9 to 7.1)
Mean hourly dose of RAC > − 0.05		3.0 (1.5 to 6.0)
6- to 12-month GOSE		4 (2 to 6)
Number alive—6 to 12 months		155
Number dead—6 to 12 months		49
Number favorable outcome—6 to 12 months (GOSE 5 to 8)		109
Number unfavorable outcome—6 to 12 months (GOSE 1 to 4)		96

*AMP* pulse amplitude of ICP, *CPP* cerebral perfusion pressure, *GCS* Glasgow Coma Score, *GOSE* Glasgow Outcome Score, *ICP* intra-cranial pressure, *IQR* inter-quartile range, *MAP* mean arterial pressure, *mmHg* millimeters of mercury, *PAx* pulse amplitude index (correlation between AMP and MAP), *PRx* pressure reactivity index (correlation between ICP and MAP), *RAC* correlation between AMP and CPP

**Table 2:** Alive/dead dichotomized groups—Mann-U and Chi-square comparison between groups

		Mean/Median (± sd or IQR)		*p* value
		Alive	Dead	
Number of patients		155	49	
Age (years)		43.5 (18.4)	56.6 (18.7)	*< 0.0001*
Sex	Male	128	35	0.089
	Female	27	13	
Admission GCS (total)		7 (5 to 13)	8 (3 to 13)	0.707
Admission GCS motor		4 (2 to 5)	4 (1 to 6)	0.863
Duration of high-frequency physiologic recording (hours)		164.1 (118.5)	145.1 (96.6)	0.472
ICP (mmHg)		12.2 (5.9)	19.9 (15.6)	*0.028*
CPP (mmHg)		71.1 (8.8)	66.6 (17.5)	0.583
% Time with ICP > 20 mmHg		10.7 (18.5)	32.4 (35.5)	*0.025*
% Time with ICP > 22 mmHg		7.1 (15.2)	27.6 (34.0)	*0.012*
Mean PRx		0.017 (0.150)	0.226 (0.269)	*< 0.0001*
Mean PAx		− 0.064 (0.175)	0.126 (0.223)	*< 0.0001*
Mean RAC		− 0.347 (0.241)	− 0.114 (0.276)	*< 0.0001*
% Time with PRx > 0		50.2 (16.7)	66.6 (20.5)	*< 0.0001*
% Time with PRx > + 0.25		28.5 (14.1)	48.0 (25.0)	*< 0.0001*
% Time with PRx > + 0.35		21.4 (12.2)	41.0 (25.7)	*< 0.0001*
% Time with PAx > 0		42.7 (18.7)	59.8 (20.9)	*< 0.0001*
% Time with PAx > + 0.25		21.6 (14.6)	39.6 (22.9)	*< 0.0001*
% Time with RAC > − 0.10		27.7 (20.1)	46.8 (23.7)	*< 0.0001*
% Time with RAC > − 0.05		24.6 (18.8)	43.3 (23.5)	*< 0.0001*
Mean hourly dose of PRx > 0		9.1 (4.6)	15.7 (11.9)	*0.001*
Mean hourly dose of PRx > + 0.25		3.9 (2.6)	8.7 (8.8)	*0.001*
Mean hourly dose of PRx > + 0.35		2.6 (1.9)	6.6 (7.5)	*0.001*
Mean hourly dose of PAx > 0		6.7 (4.3)	12.1 (8.9)	*0.001*
Mean hourly dose of PAx > + 0.25		2.5 (2.2)	6.0 (5.9)	*< 0.0001*
Mean hourly dose of RAC > − 0.10		4.4 (4.2)	9.6 (8.3)	*< 0.0001*
Mean hourly dose of RAC > − 0.05		3.8 (3.7)	8.5 (7.7)	*< 0.0001*

*AMP* pulse amplitude of ICP, *CPP* cerebral perfusion pressure, *GCS* Glasgow coma score, *GOSE* Glasgow outcome score, *ICP* intra-cranial pressure, *IQR* inter-quartile range, *MAP* mean arterial pressure, *mmHg* millimeters of mercury, *PAx* pulse amplitude index (correlation between AMP and MAP), *PRx* pressure reactivity index (correlation between ICP and MAP), *RAC* correlation between AMP and CPP, *sd* standard deviation

Italicized *p* values are those reaching statistical significance (i.e., *p* < 0.05)

**Table 3:** Favorable/unfavorable dichotomized groups—Mann-U and Chi-square comparison between groups

		Mean/median (± sd or IQR)		*p* value
		Favorable	Unfavorable	
*Number of patients*		95	109	
*Age (years)*		40.9 (17.3)	51.6 (19.6)	*< 0.0001*
*Sex*	*Male*	85	78	0.463
	*Female*	10	31	
*Admission GCS (total)*		8 (6 to 13)	7 (4 to 11)	0.136
*Admission GCS motor*		5 (3 to 6)	4 (1 to 6)	0.110
*Duration of high-frequency physiologic recording (hours)*		151.6 (119.1)	166.5 (108.8)	0.125
*ICP (mmHg)*		12.7 (6.6)	15.3 (11.7)	0.876
*CPP (mmHg)*		71.0 (8.9)	68.8 (13.6)	1.000
*% Time with ICP > 20 mmHg*		11.0 (19.1)	20.3 (29.2)	0.482
*% Time with ICP > 22 mmHg*		7.4 (16.3)	16.0 (26.7)	0.310
*Mean PRx*		0.006 (0.153)	0.121 (0.230)	*0.001*
*Mean PAx*		− 0.082 (0.173)	0.036 (0.219)	*0.001*
*Mean RAC*		− 0.387 (0.233)	− 0.207 (0.269)	*< 0.0001*
*% Time with PRx > 0*		49.1 (16.9)	58.6 (19.7)	*0.002*
*% Time with PRx > + 0.25*		27.5 (14.4)	38.1 (21.4)	*0.001*
*% Time with PRx > + 0.35*		20.6 (12.5)	30.9 (21.2)	*0.001*
*% Time with PAx > 0*		40.8 (18.4)	52.1 (21.0)	*0.001*
*% Time with PAx > + 0.25*		20.1 (14.2)	31.0 (20.3)	*< 0.0001*
*% Time with RAC >* − *0.10*		24.4 (18.6)	39.1 (23.4)	*< 0.0001*
*% Time with RAC >* − *0.05*		21.7 (17.4)	35.6 (22.8)	*< 0.0001*
*Mean hourly dose of PRx > 0*		8.7 (4.6)	12.5 (9.1)	*0.003*
*Mean hourly dose of PRx > + 0.25*		3.7 (2.6)	6.3 (6.6)	*0.001*
*Mean hourly dose of PRx > + 0.35*		2.4 (1.9)	4.6 (5.5)	*0.001*
*Mean hourly dose of PAx > 0*		6.1 (4.1)	9.6 (7.2)	*0.001*
*Mean hourly dose of PAx > + 0.25*		2.3 (2.1)	4.3 (4.5)	*< 0.0001*
*Mean hourly dose of RAC >* − *0.10*		3.7 (3.4)	7.4 (7.0)	*< 0.0001*
*Mean hourly dose of RAC >* − *0.05*		3.1 (2.9)	6.4 (6.4)	*< 0.0001*

*AMP* pulse amplitude of ICP, *CPP* cerebral perfusion pressure, *GCS* Glasgow coma score, *GOSE* Glasgow outcome score, *ICP* intra-cranial pressure, *IQR* inter-quartile range, *MAP* mean arterial pressure, *mmHg* millimeters of mercury, *PAx* pulse amplitude index (correlation between AMP and MAP), *PRx* pressure reactivity index (correlation between ICP and MAP), *RAC* correlation between AMP and CPP, *sd* standard deviation

Italicized *p* values are those reaching statistical significance (i.e., *p* < 0.05)

### Comparison between dichotomized outcome groups

Identical statistically significant differences between dichotomized outcome groups were noted in both the total population and the non-DC cohort (see Tables 3, 4 and Appendix A). In general, for the alive/dead outcome groups, the following were statistically higher for the death group: age, mean ICP, % time with ICP > 20 mmHg, % time with ICP > 22 mmHg, mean PRx, mean PAx, mean RAC, mean % time above all index thresholds, and mean hourly dose above all index thresholds.

**Table 4:** Univariate logistic regression analysis–total population–cerebrovascular reactivity index based measures

Model	AUC A/D (95% CI)	*p* value	AUC F/U (95% CI)	*p* value
*Mean PRx*	0.748 (0.662–x`0.834)	*< 0.0001*	0.649 (0.574–0.724)	*0.0002*
*Mean PAx*	0.739 (0.654–0.825)	*< 0.0001*	0.658 (0.583–0.732)	*< 0.0001*
*Mean RAC*	0.742 (0.661–0.823)	*< 0.0001*	0.691 (0.619–0.763)	*< 0.0001*
% Time above PRx thresholds				
*% Time above 0*	0.731 (0.643–0.818)	*< 0.0001*	0.637 (0.561–0.712)	*0.0005*
*% Time above + 0.25*	0.736 (0.648–0.824)	*< 0.0001*	0.647 (0.572–0.722)	*0.0002*
*% time above + 0.35*	0.737 (0.648–0.825)	*< 0.0001*	0.651 (0.576–0.726)	*0.0002*
% Time above PAx threshold				
*% Time above 0*	0.729 (0.644–0.813)	*< 0.0001*	0.656 (0.581–0.730)	*0.0001*
*% Time above + 0.25*	0.741 (0.657–0.825)	*< 0.0001*	0.670 (0.596–0.743)	*< 0.0001*
% time above RAC thresholds				
*% Time above − 0.10*	0.736 (0.657–0.816)	*< 0.0001*	0.692 (0.620–0.764)	*< 0.0001*
*% Time above − 0.05*	0.738 (0.659–0.818)	*< 0.0001*	0.691 (0.619–0.763)	*< 0.0001*
Hourly dose above PRx thresholds				
*Mean dose above 0*	0.680 (0.586–0.774)	*< 0.0001*	0.631 (0.555–0.707)	*0.0009*
*Mean dose above + 0.25*	0.696 (0.604–0.787)	*< 0.0001*	0.641 (0.565–0.716)	*0.0013*
*mean dose above + 0.35*	0.701 (0.610–0.793)	*< 0.0001*	0.647 (0.572–0.722)	*0.0017*
Hourly dose above PAx thresholds				
*Mean dose above 0*	0.694 (0.602–0.785)	*< 0.0001*	0.659 (0.585–0.733)	*0.0002*
*Mean dose above + 0.25*	0.719 (0.631–0.806)	*< 0.0001*	0.675 (0.602–0.748)	*0.0004*
Hourly dose above RAC thresholds				
*Mean dose above − 0.10*	0.717 (0.635–0.800)	*< 0.0001*	0.690 (0.618–0.762)	*< 0.0001*
*Mean dose above − 0.05*	0.720 (0.637–0.803)	*< 0.0001*	0.692 (0.620–0.763)	*< 0.0001*

*A/D* alive/dead, *AMP* pulse amplitude of ICP, *AUC* area under the receiver operating curve, *CPP* cerebral perfusion pressure, *CI* confidence interval, *F/U* favorable/unfavorable outcome (i.e., favorable = Glasgow outcome scale of 5 to 8; unfavorable = Glasgow outcome scale of 1 to 4), *ICP* intra-cranial pressure, *IMPACT* International Mission for Prognosis and Analysis of Clinical Trials, *MAP* mean arterial pressure, *PAx* pulse amplitude index (correlation between AMP and MAP), *PRx* pressure reactivity index (correlation between ICP and MAP), *RAC* correlation between AMP and CPP

CORE model consisted of age, admission Glasgow coma scale motor score, and pupil response (normal bilaterally, unilateral unreactive, or bilaterally unreactive)

Italicized *p* values are those reaching statistical significance (i.e., *p* < 0.05)

Comparing favorable/unfavorable outcome groups, the statistically significant differences in variables were the same, with the exception for ICP-based variables (mean ICP, % time with ICP > 20 mmHg and > 22 mmHg), where these were not significantly different between groups.

### Univariate logistic regression analysis

Exploring just the ICP-based cerebrovascular reactivity index variables (i.e., mean index value, % time above threshold, and mean hourly dose above threshold) and their associations with dichotomize outcomes, identical results were seen for both the total population and the non-DC cohort. Overall, the AUCs for RAC-based variables were higher in association with both alive/dead and favorable/unfavorable outcomes, as displayed in Table 4 (total population) and Appendix B of the supplementary materials (non-DC cohort). However, comparing AUCs, there was no statistically significant difference between PRx, PAx, or RAC, when comparing similar variables (i.e., mean value, % time above thresholds, hourly dose above threshold). Thus, when assessing the core indices for outcome association, all three perform similarly, with RAC trending towards higher AUCs.

## Discussion

Using the high-resolution ICU cohort from the multi-center CENTER-TBI study, we have performed a basic analysis of the outcome association of the three ICP-derived cerebrovascular reactivity indices (PRx, PAx, and RAC) in adult moderate/severe TBI. Some interesting results deserve highlighting.

Using both the total patient population and those without DC, all ICP-derived indices performed similarly in their outcome association capacity for both 6- to 12-month dichotomized outcomes. The variables derived from the indices and their known critical thresholds were all statistically significantly associated with both outcomes of interest. RAC variables (i.e., mean RAC, % time above threshold, and mean hourly dose of RAC above threshold) overall displayed the highest AUCs compared to those similar variables derived from PRx and PAx. This, however, was not statically different when comparing the AUC’s via Delong’s test. Thus, all core index variables performed similarly in outcome association, with a trend to higher AUCs for RAC. These results are in keeping with a previous large retrospective cohort analysis conducted to evaluate these three indices [22]. This lack of statistical significance between AUCs may be a function of the relatively lower patient numbers, 204 for total population and 159 for non-DC cohort. Previous retrospective single-center comparisons were based on much larger cohorts (*n* = 358) [7, 18, 22]. Further, larger studies in patients with this type of high-resolution data is required to confirm credibility of our results. Such larger multi-center studies are in the planning phase and not currently underway. However, when such studies are running, it will be imperative to ensure that such complex high-frequency physiologic data is curated in such a way that allows for sharing between centers globally. It is only through such global initiatives, with standards in complex data collection/curation, that we will be able to produce large enough data sets to begin to definitively answer our questions related to metrics derived for high-frequency digital physiologic information.

Comparing all three ICP-derived indices of cerebrovascular reactivity, they all performed similarly in their association with both dichotomized outcomes. This is in keeping with the only other study published evaluating all three indices in a single population [22]. As such, these findings were not a surprise, but confirmatory. There was a trend, as mentioned, for AUCs related to RAC to be higher. This was also seen in the previous retrospective work [22]. Thus, the next question may be why? All three indices have been validated as measures of the LLA in experimental models of arterial hypotension and/or intra-cranial hypertension [4, 19, 23]. Therefore, it is not surprising that they carry similar information regarding vascular reactivity, and thus perform similarly in clinical studies when assessing outcome prediction. It is currently unknown if one index is superior to another in adult TBI. This would require much larger high-resolution patient populations, allowing various sub-population analyses to be performed, including but not limiting such sub-population analyses to those with persistently low ICP, persistently high ICP, those with craniectomy (both primary and secondary), those with various types of intra-cranial pathology (i.e., for example patients with contusions versus diffuse axonal injury, etc), variation in systemic fluid volume, and those with various types of ICU therapies on board (i.e., sedatives, barbiturates, vasopressors, CSF drainage, and hypothermia). As such, at this time, it remains uncertain which index is superior in adult TBI.

Finally, strictly evaluating the difference in mean values for variables between both dichotomized groups, it is clear that the cerebrovascular reactivity indices are far worse in those with poorer outcomes. The mean values, % time above threshold, and hourly dose above threshold were statistically higher for both those patients who died and had unfavorable outcomes. This is in contrast to the ICP variables (i.e., % time with ICP > 20 mmHg and % time with ICP > 22 mmHg), where the percentage of time above ICP threshold appears to only be significantly different (i.e., higher) in those patients whom died, without any statistically significant differences for these ICP variables noted between favorable and unfavorable dichotomized outcome groups. This highlights the known strong relationship between elevated ICP and death in TBI, with a weaker relation between ICP and long-term functional outcome [5, 17, 18]. The results from this study suggest that ICP-derived indices of cerebrovascular reactivity may be better predictors of functional outcome in adult TBI, compared to ICP values alone, emphasizing the potential importance of cerebrovascular reactivity monitoring in prognostication. However, the results of this study are preliminary, with these relationships requiring much further investigation.

### Limitations

Despite the interesting and significant results, there are limitations which require addressing.

First, despite having prospective multi-center data, the overall patient numbers for the total and non-DC cohorts are quite low. This may impact the lack of statistical significance between AUCs found when comparing PRx, PAx, and RAC. Thus, the results here are only preliminary and require further validation with larger high-resolution data sets gathered in a multi-center fashion. This power issue may be further reflected in the inability to display any statistically significant differences between admission GCS and the dichotomized outcome groups. This particular finding was slightly surprising, given that many other studies have documented worse admission GCS is associated with worse outcomes at 6 to 12 months post-TBI [7, 14]. This lack of difference is likely secondary to small patient numbers in the high-resolution ICU cohort, given the complexity in data curation for this cohort. It is likely that when future analysis of the entire CENTER-TBI ICU cohort occurs, where there is no high-frequency physiology recorded, that GCS will display significant differences between dichotomized outcome groups.

Second, treatment heterogeneity may have played a role in the signal values and associations seen. CENTER TBI was a prospective observational study, as such there exists a potential for intra-center and inter-center treatment heterogeneity. In particular, this study was an observational, not interventional, study, with the individual centers providing care a level consistent with BTF and local standards. As such, the heterogeneity in the extent and aggressiveness of care may have varied between centers. The aggressiveness of care would impact the duration of monitoring data obtained, with more aggressive longer-term treatments leading to extended duration recordings. As such, this treatment heterogeneity may not only impact the recorded physiologic signals, secondary to the therapies applied to ICP elevations, but also the duration of the recordings obtained, and thus potentially the associations with the dichotomized outcomes seen in our study. With that said, all other large retrospective studies on cerebrovascular reactivity in the past have been subjected to such limitations of purely observational data, with their results in parallel to those found in the current study [7, 18, 22].

Third, the evaluation between systemic volume status, such as measured through central venous pressure or other methodologies, may prove interesting when compared to cerebrovascular reactivity monitoring. There is the potential that fluid volume may correlate with vascular reactivity as measured through ICP-derived indices and requires investigation in future larger studies.

Fourth, the population chosen was that with an outcome recorded at 6 to 12 months and high-frequency digital signals, requiring all patients to have these particular set of variables for the models, hence the low patient numbers overall. As such, we utilized the available non-imputed data and focused only on univariate models comparing various variables to dichotomized outcomes. The focus of this manuscript was on univariate associations with global outcome, in order to provide validation for previous retrospective single-center works on the topic, [22] while providing insight into these measures for future work on multi-variable prognostication that will be part of other works from the CENTER-TBI high-resolution sub-study. The potential exists for these relationships to become insignificant when large multi-variable models are assessed. At the time of this manuscript composition, there is currently a separate larger project underway for imputation of missing data components across all cohorts in CENTER-TBI (high resolution and non-high resolution), the results of which are not currently available and will be published in a separate piece. Thus, at this time, all we can comment on are the strength of univariate associations with outcome, though much further work on the topic is planned as data becomes available.

Finally, it must be re-emphasized that the results here are preliminary only given the relatively small patient numbers and limitations outlined. As such, they require much further investigation and validation.

## Conclusion

PRx, PAx, and RAC appear similar in their associations with 6- to 12-month outcome in moderate/severe adult TBI, with RAC trending towards higher AUC values. Further work is required to determine the exact role for each of these cerebrovascular indices in monitoring TBI patients.

### Electronic supplementary material

ESM 1 (DOCX 26 kb)

ESM 2 (DOCX 16 kb)

## Appendix

**CENTER-TBI High Resolution Sub-Study Participants and Investigators:**

Audny Anke^1^, Ronny Beer^2^, Bo-Michael Bellander^3^, Andras Buki^4^, Giorgio Chevallard^5^, Arturo Chieregato^5^, Giuseppe Citerio^6,7^, Endre Czeiter^8^, Bart Depreitere^9^, George Eapen^†^, Shirin Frisvold^10^, Raimund Helbok^2^, Stefan Jankowski^11^, Daniel Kondziella^12^, Lars-Owe Koskinen^13^, Geert Meyfroidt^14^, Kirsten Moeller^15^, David Nelson^3^, Anna Piippo-Karjalainen^16^, Andreea Radoi^17^, Arminas Ragauskas^18^, Rahul Raj^16^, Jonathan Rhodes^19^, Saulius Rocka^18^, Rolf Rossaint^20^, Juan Sahuquillo^17^, Oliver Sakowitz^21,22^, Ana Stevanovic^20^, Nina Sundström^23^, Riikka Takala^24^, Tomas Tamosuitis^25^, Olli Tenovuo^26^, Peter Vajkoczy^27^, Alessia Vargiolu^6^, Rimantas Vilcinis^28^, Stefa Wolf^29^, Alexander Younsi^22^

^1^Department of Physical Medicine and Rehabilitation, University hospital Northern Norway

^2^Department of Neurology, Neurological Intensive Care Unit, Medical University of Innsbruck, Innsbruck, Austria

^3^Department of Neurosurgery & Anesthesia & intensive care medicine, Karolinska University Hospital, Stockholm, Sweden

^4^Department of Neurosurgery, University of Pecs and MTA-PTE Clinical Neuroscience MR Research Group and Janos Szentagothai Research Centre, University of Pecs, Hungarian Brain Research Program, Pecs, Hungary

^5^NeuroIntensive Care, Niguarda Hospital, Milan, Italy

^6^NeuroIntensive Care Unit, Department of Anesthesia & Intensive Care, ASST di Monza, Monza, Italy

^7^School of Medicine and Surgery, Università Milano Bicocca, Milano, Italy

^8^Department of Neurosurgery, University of Pecs and MTA-PTE Clinical Neuroscience MR Research Group and Janos Szentagothai Research Centre, University of Pecs, Hungarian Brain Research Program (Grant No. KTIA 13 NAP-A-II/8), Pecs, Hungary

^9^Department of Neurosurgery, University Hospitals Leuven, Leuven, Belgium

^10^Department of Anesthesiology and Intensive care, University Hospital Northern Norway, Tromso, Norway

^11^Neurointensive Care, Sheffield Teaching Hospitals NHS Foundation Trust, Sheffield, UK

^12^Departments of Neurology, Clinical Neurophysiology and Neuroanesthesiology, Region Hovedstaden Rigshospitalet, Copenhagen, Denmark

^13^Department of Clinical Neuroscience, Neurosurgery, Umea University Hospital, Umea, Sweden

^14^Intensive Care Medicine, University Hospitals Leuven, Leuven, Belgium

^15^Department Neuroanesthesiology, Region Hovedstaden Rigshospitalet, Copenhagen, Denmark

^16^Helsinki University Central Hospital, Helsinki, Finland

^17^Department of Neurosurgery, Vall d'Hebron University Hospital, Barcelona, Spain

^18^Department of Neurosurgery, Kaunas University of technology and Vilnius University, Vilnius, Lithuania

^19^Department of Anesthesia, Critical Care & Pain Medicine NHS Lothian & University of Edinburg, Edinburgh, UK

^20^Department of Anaesthesiology, University Hospital of Aachen, Aachen, Germany

^21^Klinik für Neurochirurgie, Klinikum Ludwigsburg, Ludwigsburg, Germany

^22^Department of Neurosurgery, University Hospital Heidelberg, Heidelberg, Germany

^23^Department of Radiation Sciences, Biomedical Engineering, Umea University Hospital, Umea, Sweden

^24^Perioperative Services, Intensive Care Medicine, and Pain Management , Turku University Central Hospital and University of Turku, Turku, Finland

^25^Neuro-intensive Care Unit, Kaunas University of Health Sciences, Kaunas, Lithuania

^26^Rehabilitation and Brain Trauma, Turku University Central Hospital and University of Turku, Turku, Finland

^27^Neurologie, Neurochirurgie und Psychiatrie, Charité – Universitätsmedizin Berlin, Berlin, Germany

^28^Department of Neurosurgery, Kaunas University of Health Sciences, Kaunas, Lithuania

^29^Interdisciplinary Neuro Intensive Care Unit , Charité – Universitätsmedizin Berlin, Berlin, Germany
