# Supplementary material for: Univariate comparison of performance of different cerebrovascular reactivity indices for outcome association in adult TBI: a CENTER-TBI study
Source: Acta Neurochir (Wien). 2019 Mar 15;161(6):1217–27. doi: 10.1007/s00701-019-03844-1 (PMC6525666; doi:10.1007/s00701-019-03844-1)
Supplement: Supplementary file 1 — (DOCX 26 kb) [file 701_2019_3844_MOESM1_ESM.docx]

Appendix A: Non-DC Population – Patient Demographics and Comparison of Variables Between Dichotomized Outcome Groups

1. Entire Population Demographics

Patient Demographics – Total non-DC Population

|  | | **Median (IQR)** |
| --- | --- | --- |
| ***Number of Patients*** | | 159 |
| ***Age (years)*** | | 50.0 (30.8 to 64.0) |
| ***Sex*** | ***Male*** | 123 |
|  | ***Female*** | 36 |
| ***Admission GCS (Total)*** | | 8 (5 to 13) |
| ***Admission GCS Motor*** | | 5 (2 to 6) |
| ***Duration of High Frequency Physiologic Recording (hours)*** | | 125.5 (87.6 to 165.4) |
| ***ICP (mm Hg)*** | | 12.3 (9.3 to 15.7) |
| ***CPP (mm Hg)*** | | 70.3 (63.9 to 77.4) |
| ***% Time with ICP > 20 mm Hg*** | | 4.6 (1.0 to 16.6) |
| ***% Time with ICP > 22 mm Hg*** | | 2.7 (0.6 to 10.0) |
| ***Mean PRx*** | | 0.034 (-0.068 to 0.172) |
| ***Mean PAx*** | | -0.023 (-0.172 to 0.098) |
| ***Mean RAC*** | | -0.346 (-0.520 to -0.165) |
| ***% Time with PRx > 0*** | | 52.2 (41.0 to 67.9) |
| ***% Time with PRx > +0.25*** | | 26.8 (19.2 to 42.5) |
| ***% Time with PRx > +0.35*** | | 20.7 (14.0 to 31.8) |
| ***% Time with PAx > 0*** | | 46.5 (30.6 to 62.7) |
| ***% Time with PAx > +0.25*** | | 21.6 (12.6 to 36.0) |
| ***% Time with RAC > -0.10*** | | 22.9 (12.0 to 41.3) |
| ***% Time with RAC > -0.05*** | | 20.3 (10.4 to 37.5) |
| ***Mean Hourly Dose of PRx > 0*** | | 8.8 (6.4 to 12.8) |
| ***Mean Hourly Dose of PRx > +0.25*** | | 3.7 (2.5 to 5.8) |
| ***Mean Hourly Dose of PRx > +0.35*** | | 2.4 (1.5 to 3.9) |
| ***Mean Hourly Dose of PAx > 0*** | | 6.6 (4.1 to 10.2) |
| ***Mean Hourly Dose of PAx > +0.25*** | | 2.4 (1.2 to 3.9) |
| ***Mean Hourly Dose of RAC > -0.10*** | | 3.4 (1.6 to 6.7) |
| ***Mean Hourly Dose of RAC > -0.05*** | | 2.7 (1.3 to 5.6) |
| ***6 to 12 Month GOSE*** | | 5 (2 to 5) |
| ***Number Alive – 6 to 12 Months*** | | 121 |
| ***Number Dead – 6 to 12 Months*** | | 38 |
| ***Number Favourable Outcome – 6 to 12 Months (GOSE 5 to 8)*** | | 82 |
| ***Number Unfavourable Outcome – 6 to 12 Months (GOSE 1 to 4)*** | | 77 |

AMP = pulse amplitude of ICP, CPP = cerebral perfusion pressure, DC = decompressive craniectomy, GCS = Glasgow Coma Score, GOSE = Glasgow Outcome Score, ICP = intra-cranial pressure, IQR = inter-quartile range, MAP = mean arterial pressure, mm Hg = millimeters of mercury, PAx = pulse amplitude index (correlation between AMP and MAP), PRx = pressure reactivity index (correlation between ICP and MAP), RAC = correlation between AMP and CPP.

1. Comparison of Demographics and Physiologic Variables Between Dichotomized Outcome Groups – Alive/Dead Dichotomization

Alive/Dead Dichotomized Groups – non-DC cohort - Mann-U and Chi-Square Comparison Between Groups

|  | | **Mean/Median (+/-sd or IQR)** | | **p-value** |
| --- | --- | --- | --- | --- |
|  |  | **Alive** | **Dead** |  |
| ***Number of Patients*** | | 121 | 38 |  |
| ***Age (years)*** | | 44.8 (18.6) | 59.0 (18.9) | **0.001** |
| ***Sex*** | ***Male*** | 97 | 26 | 0.131 |
|  | ***Female*** | 24 | 12 |  |
| ***Admission GCS (Total)*** | | 8 (5 to 13) | 9 (4 to 13) | 0.530 |
| ***Admission GCS Motor*** | | 5 (3 to 6) | 4 (1 to 6) | 0.592 |
| ***Duration of High Frequency Physiologic Recording (hours)*** | | 156.6 (114.5) | 131.2 (85.1) | 0.264 |
| ***ICP (mm Hg)*** | | 12.2 (6.1) | 21.0 (16.4) | **0.012** |
| ***CPP (mm Hg)*** | | 70.9 (9.4) | 65.5 (17.4) | 0.455 |
| ***% Time with ICP > 20 mm Hg*** | | 9.7 (16.7) | 36.9 (36.3) | **0.002** |
| ***% Time with ICP > 22 mm Hg*** | | 6.4 (14.2) | 31.8 (35.2) | **0.001** |
| ***Mean PRx*** | | 0.020 (0.152) | 0.235 (0.292) | **0.001** |
| ***Mean PAx*** | | -0.061 (0.283) | 0.138 (0.237) | **<0.0001** |
| ***Mean RAC*** | | -0.378 (0.249) | -0.141 (0.284) | **<0.0001** |
| ***% Time with PRx > 0*** | | 65.4 (16.7) | 66.8 (22.4) | **0.001** |
| ***% Time with PRx > +0.25*** | | 28.8 (14.4) | 48.5 (26.9) | **0.001** |
| ***% Time with PRx > +0.35*** | | 21.8 (12.6) | 41.6 (27.6) | **0.001** |
| ***% Time with PAx > 0*** | | 43.1 (19.7) | 61.0 (21.6) | **<0.0001** |
| ***% Time with PAx > +0.25*** | | 22.3 (15.6) | 41.3 (23.1) | **<0.0001** |
| ***% Time with RAC > -0.10*** | | 25.5 (20.7) | 44.9 (24.8) | **<0.0001** |
| ***% Time with RAC > -0.05*** | | 22.9 (19.5) | 41.7 (22.5) | **<0.0001** |
| ***Mean Hourly Dose of PRx > 0*** | | 9.2 (4.7) | 16.0 (13.2) | **0.014** |
| ***Mean Hourly Dose of PRx > +0.25*** | | 4.0 (2.7) | 9.1 (9.7) | **0.009** |
| ***Mean Hourly Dose of PRx > +0.35*** | | 2.7 (2.0) | 6.9 (8.2) | **0.008** |
| ***Mean Hourly Dose of PAx > 0*** | | 6.8 (4.5) | 12.5 (9.3) | **0.001** |
| ***Mean Hourly Dose of PAx > +0.25*** | | 2.6 (2.4) | 6.3 (6.0) | **<0.0001** |
| ***Mean Hourly Dose of RAC > -0.10*** | | 4.1 (4.4) | 9.2 (8.3) | **0.001** |
| ***Mean Hourly Dose of RAC > -0.05*** | | 3.5 (3.9) | 8.2 (7.6) | **0.001** |

AMP = pulse amplitude of ICP, CPP = cerebral perfusion pressure, GCS = Glasgow Coma Score, GOSE = Glasgow Outcome Score, ICP = intra-cranial pressure, IQR = inter-quartile range, MAP = mean arterial pressure, mm Hg = millimeters of mercury, PAx = pulse amplitude index (correlation between AMP and MAP), PRx = pressure reactivity index (correlation between ICP and MAP), RAC = correlation between AMP and CPP, sd = standard deviation. Bolded p-values are those reaching statistical significance (ie. p<0.05).

1. Comparison of Demographics and Physiologic Variables Between Dichotomized Outcome Groups – Favourable/Unfavourable Dichotomization

Favourable/Unfavourable Dichotomized Groups – non-DC cohort - Mann-U and Chi-Square Comparison Between Groups

|  | | **Mean/Median (+/-sd or IQR)** | | **p-value** |
| --- | --- | --- | --- | --- |
|  |  | **Favourable** | **Unfavourable** |  |
| ***Number of Patients*** | | 82 | 77 |  |
| ***Age (years)*** | | 41.9 (17.3) | 54.8 (19.7) | **<0.0001** |
| ***Sex*** | ***Male*** | 66 | 57 | 0.331 |
|  | ***Female*** | 16 | 20 |  |
| ***Admission GCS (Total)*** | | 8 (6 to 13) | 8 (4 to 13) | 0.231 |
| ***Admission GCS Motor*** | | 5 (4 to 6) | 4 (2 to 6) | 0.159 |
| ***Duration of High Frequency Physiologic Recording (hours)*** | | 150.3 (123.5) | 150.6 (92.5) | 0.427 |
| ***ICP (mm Hg)*** | | 12.6 (6.8( | 16.1 (12.8) | 0.337 |
| ***CPP (mm Hg)*** | | 71.0 (9.5) | 68.2 (14.1) | 0.566 |
| ***% Time with ICP > 20 mm Hg*** | | 10.2 (17.7) | 22.6 (30.8) | 0.190 |
| ***% Time with ICP > 22 mm Hg*** | | 6.9 (15.7) | 18.4 (28.8) | 0.100 |
| ***Mean PRx*** | | 0.009 (0.156) | 0.137 (0.248) | **0.002** |
| ***Mean PAx*** | | -0.075 (0.176) | 0.053 (0.232) | **0.001** |
| ***Mean RAC*** | | -0.402 (0.237) | -0.235 (0.290) | **0.001** |
| ***% Time with PRx > 0*** | | 49.4 (17.2) | 59.6 (20.5) | **0.004** |
| ***% Time with PRx > +0.25*** | | 27.9 (14.9) | 39.5 (22.8) | **0.004** |
| ***% Time with PRx > +0.35*** | | 21.0 (13.0) | 32.4 (22.9) | **0.003** |
| ***% Time with PAx > 0*** | | 41.4 (18.9) | 53.7 (22.3) | **0.001** |
| ***% Time with PAx > +0.25*** | | 20.9 (14.9) | 33.3 (21.6) | **0.001** |
| ***% Time with RAC > -0.10*** | | 23.6 (18.9) | 37.2 (25.3) | **0.001** |
| ***% Time with RAC > -0.05*** | | 21.0 (17.8) | 34.1 (24.6) | **0.001** |
| ***Mean Hourly Dose of PRx > 0*** | | 8.8 (4.7) | 13.0 (10.2) | **0.010** |
| ***Mean Hourly Dose of PRx > +0.25*** | | 3.8 (2.7) | 6.8 (7.4) | **0.009** |
| ***Mean Hourly Dose of PRx > +0.35*** | | 2.5 (2.0) | 5.0 (6.2) | **0.008** |
| ***Mean Hourly Dose of PAx > 0*** | | 6.3 (4.3) | 10.2 (7.7) | **0.001** |
| ***Mean Hourly Dose of PAx > +0.25*** | | 2.4 (2.2) | 4.7 (4.8) | **0.001** |
| ***Mean Hourly Dose of RAC > -0.10*** | | 3.6 (3.5) | 7.2 (7.3) | **0.001** |
| ***Mean Hourly Dose of RAC > -0.05*** | | 3.1 (3.0) | 6.3 (6.7) | **0.001** |

AMP = pulse amplitude of ICP, CPP = cerebral perfusion pressure, GCS = Glasgow Coma Score, GOSE = Glasgow Outcome Score, ICP = intra-cranial pressure, IQR = inter-quartile range, MAP = mean arterial pressure, mm Hg = millimeters of mercury, PAx = pulse amplitude index (correlation between AMP and MAP), PRx = pressure reactivity index (correlation between ICP and MAP), RAC = correlation between AMP and CPP, sd = standard deviation. Bolded p-values are those reaching statistical significance (ie. p<0.05).
