# Supplementary material for: Univariate comparison of performance of different cerebrovascular reactivity indices for outcome association in adult TBI: a CENTER-TBI study
Source: Acta Neurochir (Wien). 2019 Mar 15;161(6):1217–27. doi: 10.1007/s00701-019-03844-1 (PMC6525666; doi:10.1007/s00701-019-03844-1)
Supplement: Supplementary file 2 — (DOCX 16 kb) [file 701_2019_3844_MOESM2_ESM.docx]

Appendix B: Univariate Logistic Regression Analysis – non-DC cohort - Cerebrovascular Reactivity Index Based Measures

| **Model** | **AUC A/D (95% CI)** | **p-value** | **AUC F/U (95% CI)** | **p-value** |
| --- | --- | --- | --- | --- |
| ***Mean PRx*** | 0.729 (0.623-0.835) | **<0.0001** | 0.653 (0.567-0.739) | **0.0004** |
| ***Mean PAx*** | 0.746 (0.650-0.842) | **<0.0001** | 0.665 (0.580-0.750) | **0.0003** |
| ***Mean RAC*** | 0.743 (0.650-0.836) | **<0.0001** | 0.663 (0.579-0.748) | **0.0003** |
| **% Time Above PRx Thresholds** | | | | |
| ***% Time Above 0*** | 0.719 (0.612-0.826) | **<0.0001** | 0.642 (0.557-0.729) | **0.0013** |
| ***% Time Above +0.25*** | 0.705 (0.596-0.814) | **<0.0001** | 0.648 (0.561-0.734) | **0.0005** |
| ***% Time Above +0.35*** | 0.706 (0.597-0.815) | **<0.0001** | 0.650 (0.564-0.736) | **0.0006** |
| **% Time Above PAx Threshold** | | | | |
| ***% Time Above 0*** | 0.732 (0.637-0.828) | **<0.0001** | 0.662 (0.577-0.747) | **0.0005** |
| ***% Time Above +0.25*** | 0.751 (0.659-0.844) | **<0.0001** | 0.678 (0.594-0.762) | **0.0002** |
| **% Time Above RAC Thresholds** | | | | |
| ***% Time Above -0.10*** | 0.734 (0.643-0.824) | **<0.0001** | 0.664 (0.580-0.747) | **0.0004** |
| ***% Time Above -0.05*** | 0.735 (0.645-0.825) | **<0.0001** | 0.664 (0.580-0.747) | **0.0004** |
| **Hourly Dose Above PRx Thresholds** | | | | |
| ***Mean Dose Above 0*** | 0.639 (0.525-0.755) | **0.0003** | 0.624 (0.536-0.712) | **0.0029** |
| ***Mean Dose Above +0.25*** | 0.654 (0.543-0.766) | **0.0004** | 0.630 (0.543-0.718) | **0.0039** |
| ***Mean Dose Above +0.35*** | 0.661 (0.551-0.772) | **<0.0001** | 0.636 (0.548-0.723) | **0.0048** |
| **Hourly Dose Above PAx Thresholds** | | | | |
| ***Mean Dose Above 0*** | 0.695 (0.591-0.799) | **<0.0001** | 0.662 (0.578-0.747) | **0.0005** |
| ***Mean Dose Above +0.25*** | 0.684 (0.634-0.824) | **<0.0001** | 0.729 (0.602-0.767) | **0.0008** |
| **Hourly Dose Above RAC Thresholds** | | | | |
| ***Mean Dose Above -0.10*** | 0.719 (0.625-0.812) | **<0.0001** | 0.671 (0.589-0.754) | **0.0005** |
| ***Mean Dose Above -0.05*** | 0.723 (0.630-0.815) | **<0.0001** | 0.673 (0.590-0.756) | **0.0006** |

A/D = alive/dead, AMP = pulse amplitude of ICP, AUC = area under the receiver operating curve, CPP = cerebral perfusion pressure, CI = confidence interval, DC = decompressive craniectomy, F/U = Favourable/Unfavourable outcome (ie. Favourable = Glasgow Outcome Scale of 5 to 8; Unfavourable = Glasgow Outcome Scale of 1 to 4), ICP = intra-cranial pressure, IMPACT = International Mission for Prognosis and Analysis of Clinical Trials, MAP = mean arterial pressure, PAx = pulse amplitude index (correlation between AMP and MAP), PRx = pressure reactivity index (correlation between ICP and MAP), RAC = correlation between AMP and CPP. CORE model consisted of age, admission Glasgow Coma Scale motor score and pupil response (normal bilaterally, unilateral unreactive, or bilaterally unreactive). Bolded p-values are those reaching statistical significance (ie. p<0.05).
